# Phylogenetic and Genome-Wide Deep-Sequencing Analyses of Canine Parvovirus Reveal Co-Infection with Field Variants and Emergence of a Recent Recombinant Strain

**DOI:** 10.1371/journal.pone.0111779

**Published:** 2014-11-03

**Authors:** Ruben Pérez, Lucía Calleros, Ana Marandino, Nicolás Sarute, Gregorio Iraola, Sofia Grecco, Hervé Blanc, Marco Vignuzzi, Ofer Isakov, Noam Shomron, Lucía Carrau, Martín Hernández, Lourdes Francia, Katia Sosa, Gonzalo Tomás, Yanina Panzera

**Affiliations:** 1 Sección Genética Evolutiva, Instituto de Biología, Facultad de Ciencias, Universidad de la República, Montevideo, Uruguay; 2 Institut Pasteur, Viral Populations and Pathogenesis Unit, Centre National de la Recherche Scientifique, Paris, France; 3 Sackler Faculty of Medicine, Tel Aviv University, Tel Aviv, Israel; University of Nantes, France

## Abstract

Canine parvovirus (CPV), a fast-evolving single-stranded DNA virus, comprises three antigenic variants (2a, 2b, and 2c) with different frequencies and genetic variability among countries. The contribution of co-infection and recombination to the genetic variability of CPV is far from being fully elucidated. Here we took advantage of a natural CPV population, recently formed by the convergence of divergent CPV-2c and CPV-2a strains, to study co-infection and recombination. Complete sequences of the viral coding region of CPV-2a and CPV-2c strains from 40 samples were generated and analyzed using phylogenetic tools. Two samples showed co-infection and were further analyzed by deep sequencing. The sequence profile of one of the samples revealed the presence of CPV-2c and CPV-2a strains that differed at 29 nucleotides. The other sample included a minor CPV-2a strain (13.3% of the viral population) and a major recombinant strain (86.7%). The recombinant strain arose from inter-genotypic recombination between CPV-2c and CPV-2a strains within the VP1/VP2 gene boundary. Our findings highlight the importance of deep-sequencing analysis to provide a better understanding of CPV molecular diversity.

## Introduction

Members of the *Parvoviridae* family are small (18–22 nm), non-enveloped icosahedral viruses that cause a wide range of diseases in animals [1]–[3] and humans [1], [4]. Canine parvovirus (CPV) is an extremely relevant member of the *Parvoviridae* family because it is the causative agent of one of the most dangerous infectious disease in young dogs and is responsible for large numbers of animal deaths worldwide [5].

CPV has a linear single-stranded DNA (ssDNA) genome (5.2 kb) with two major open reading frames (ORFs) [6]. The left ORF encodes nonstructural proteins NS1 and NS2, which are essential for replication and DNA packaging [7]. The N-terminal regions of NS1 and NS2 are identical in sequence, whereas the C-terminal region of NS2 is derived from differential splicing of the mRNA and is translated from a different reading frame than NS1. The right ORF encodes the viral capsid proteins 1 and 2 (VP1 and VP2), which are the main antigens that induce protective antibodies [8]–[10]. VP1 and VP2 are splice variants and are identical in sequence, except for a 143-amino-acid (aa) N-terminal region that is unique to VP1. At both ends of the CPV genome, there are non-translated regions with hairpin structures that are necessary for priming replication [6].

CPV is a host-specific variant of the feline panleukopenia virus (FPV) that emerged in the 1970s because of an interspecies jump from other carnivores to dogs [11]. The newly emerged CPV, named CPV-2, rapidly spread worldwide in 1978 causing an acute enteritis disease in dogs [5]. The successful cross-species viral transfer and adaptation to the new canine host involved a small number of point mutations in the viral capsid proteins [12].

In 1979, CPV-2 was replaced globally by CPV-2a, a genetic variant that differs at five residues in VP2 and regained the ability to infect cats and other carnivores [12], [13]. CPV-2a became the new dominant lineage and underwent further evolution, retaining several point mutations. Some of these mutations change the antigenic properties of the capsid and have reached high frequencies in viral populations. In addition to the original CPV-2a antigenic type, there are two known antigenic variants, referred to as CPV-2b and CPV-2c. CPV-2b was first detected in 1984 in the United States [14], and CPV-2c was identified in 2000 in Italy [15]. Antigenic differences among the three variants are caused by changes at residue 426 (Asn in CPV-2a, Asp in CPV-2b, and Glu in CPV-2c), which is located at the top of the three-fold spike, the main antigenic domain of VP2. CPV-2a, -2b, and -2c are currently circulating worldwide, and their relative frequencies and genetic characteristics vary geographically and temporally [16]–[19].

CPV shows a high substitution rate, resembling the more rapidly evolving RNA viruses [20]. However, as a recently emerging pathogen, CPV has accumulated a relatively small number of substitutions; the FPV and CPV clades are separated by 16 nucleotide substitutions, whereas 7 substitutions separate the CPV-2 subclade from the CPV-2a subclade. The majority of these substitutions (11 between FPV and CPV and 5 between CPV-2 and CPV-2a) are located in the capsid protein gene region [19]. Because these few nucleotide changes in VP2 were sufficient to allow for host shift and adaptability, most studies have focused on the evolution of the VP2 gene. In contrast, NS genes are studied much less frequently; for example, there is not a single NS sequence of the CPV-2c variant in the GenBank database.

Although point mutation is considered the main mechanism for generating genomic diversity in CPV [19], co-infection and recombination have also been explored as variability-inducing mechanisms. Co-infection with different CPV strains has been proposed to be a source of genetic complexity and diversity in parvoviruses [21]–[24]. These studies were mainly based on sporadic cases, and the relevance and rate of co-infection remain largely unknown in natural CPV populations. Co-infection with divergent strains can result in recombination between viral genomes. Although evidence of recombination was not initially detected in a large-scale analysis of CPV genomes [19], recombination has been more recently described between the CPV vaccine (CPV-2 type) and field strains by analyzing the VP1 gene [25]. There is also evidence that CPV can recombine with the closely related carnivore parvoviruses FPV and mink enteritis virus (MEV) [26], [27]. Recombination between currently circulating field strains (i.e., CPV-2a, CPV-2c, and CPV-2b) has not yet been reported. Analysis of recombination between CPV variants is difficult, possibly because methods used to detect mosaic genomes perform poorly and fail to identify breakpoints when the parental sequences are short and have low diversity [28], [29]. In addition to the nucleotide similarity of CPV variants, which makes it difficult to show conclusively that recombination has indeed occurred, most CPV populations have well-defined geographical ranges that may preclude the co-circulation of two divergent strains, a prerequisite for detecting cases of co-infection and recombination.

During the last decade, there have been interesting changes in the distribution and variability of CPV variants in Uruguay. Until 2009, Uruguay had a homogenous CPV-2c population [30]. In 2010, a divergent Asiatic CPV-2a strain invaded the CPV-2c homogenous population and co-circulated with CPV-2c during 2010 and 2011 [31]. In the present study, we took advantage of the convergence of these divergent CPV-2c and CPV-2a strains to analyze CPV dynamics and evolution in the field. We obtained full-length genomes and performed deep sequencing of Uruguayan samples to further estimate the genetic divergence in the CPV-2a and CPV-2c variants and to explore the occurrence of co-infection and recombination events.

## Materials and Methods

### Strains

We analyzed dog fecal samples collected from 40 puppies (aged 1 to 10 months) of diverse breeds that had symptoms suggestive of parvovirus infection, and only samples that tested positive for CPV by PCR amplification of a partial VP2 sequence were used in further analyses [30], [31]. Samples were provided by veterinary clinics located in several Uruguayan regions and were collected from 2006 to 2011 (Table 1).

**Table 1:** Uruguayan dog fecal samples used in this study.

Sample	Collection year	Sampling location^a^	CPV variant	GenBank accession no
12	2006	C	2c	KM457103
47	2006	I	2c	KM457104
52	2006	P	2c	KM457105
55	2006	P	2c	KM457106
72	2007	C	2c	KM457107
82	2007	C	2c	KM457108
95	2007	P	2c	KM457109
101	2007	C	2c	KM457110
120	2008	I	2c	KM457111
135	2008	P	2c	KM457112
152	2008	P	2c	KM457113
169	2008	I	2c	KM457114
173	2009	C	2c	KM457115
185	2009	I	2c	KM457116
187	2009	P	2c	KM457117
190	2009	I	2c	KM457118
235	2010	I	2c	KM457119
242	2010	C	2c	KM457120
247	2010	I	2c	KM457121
258	2010	C	2c	KM457122
261	2010	C	2c	KM457123
243	2010	P	2a	KM457102
245	2010	C	2a	KM457132
250	2010	C	2a	KM457133
280	2010	I	2a	KM457134
307	2011	I	2c	KM457124
317	2011	C	2c	KM457125
318	2011	I	2c	KM457126
326	2011	C	2c	KM457127
346	2011	P	2c	KM457128
349	2011	P	2c	KM457129
354	2011	P	2c	KM457130
368	2011	C	2c	KM457131
370	2011	C	2a	KM457141
370	2011	C	2c	KM457142
306	2011	C	2a	KM457135
315	2011	C	2a	KM457136
344	2011	P	2a	KM457137
363	2011	P	2a	KM457138
364	2011	C	rec	KM457139
364	2011	C	2a	KM457143
365	2011	P	2a	KM457140

a (C) Capital city (Montevideo); (P) Periphery: cities/regions located in or near (within 15 km) the boundary of Montevideo; (I) Interior (remainder of the country). The VP2 gene sequences for some of these strains have been previously reported [31].

### DNA extraction, PCR, and sequencing

Viral DNA was extracted from dog feces using the QIAamp DNA Mini kit (Qiagen). Partial and full-length genome amplification was performed with a Long PCR Enzyme Mix (Thermo Scientific) using the conditions suggested by the supplier. Amplicons and primers are described in Figure S1. PCR products were either directly sequenced or cloned into bacteria after being purified with the ISOLATE II PCR and Gel kit (Bioline).

### Cloning procedure

A single amplicon (4655 bp) including the full-length CPV coding region (4269 bp) and partial 5′ and 3′ non-translated regions was cloned and sequenced at a commercial laboratory (Macrogen Inc.). Individual clones were partially (16 clones) or completely (4 clones) sequenced.

### Sequence assembly and alignment

Sequence assembly and consensus analysis were performed using the Lasergene Genomics Suite (DNASTAR). DNA sequences were aligned using MAFFT [32].

### Recombination analyses

SplitsTree4 [33] was used to infer a recombination network from full-length coding genomes of CPV strains. Phylogenetic networks are useful for identifying strains in a dataset that have conflicting ancestral relationships produced by non-vertical evolution (e.g., recombination). A Phi test value of p<0.05 was considered statistical evidence of recombination in the dataset. Identification of potential recombinant and parental sequences and localization of possible recombinant break points were performed using the RDP4 program, which implements seven distinct algorithms for characterization of recombinant sequences [34].

### Phylogenetic analyses of non-recombinant genomic regions

For DNA alignments, the best-fit model of nucleotide substitution (HKY+I) was selected under the Akaike information criterion and Bayesian information criterion in jModelTest [35].

Maximum-likelihood trees, with approximate likelihood ratio tests for the support of internal nodes, were inferred using PhyML [36]. Phylogenetic trees were visualized and edited with FigTree [37].

### Deep-sequencing analysis

DNA isolation for deep sequencing was performed using a fresh batch of dog feces. Twenty nanograms of DNA of each sample were used to generate two PCR amplicons (2255 and 2663 bp) that together span the entire CPV coding sequence. Amplicons from three independent PCR reactions were pooled and then purified using the Nucleospin Gel and PCR Clean-up kit (Macherey-Nagel), and the DNA was quantified with a Nano-drop spectrophotometer (Thermo Scientific). PCR products were then fragmented with Fragmentase (New England Biolabs), linked to Illumina multiplex adapters, and clusterized and sequenced with Illumina cBot and GAIIX technology. Quality filtering (95–98% of reads passed) and adaptor trimming were performed with fastq-mcf [38]. The 69-nucleotide (nt) reads were aligned to a CPV-2a Uruguayan sequence as a reference with a maximum of two mismatches per read and no gaps, using the Burrows-Wheeler Aligner [39]. Alignments were processed using SAMtools [40] to obtain the nucleotide/base call at each position. Nucleotide base frequency was calculated at each position as the ratio of the number of base (A, T, C or G) reads to depth of coverage.

## Results

### Strain identification

Sequences of the complete CPV coding region (4269 nt) of strains isolated from 40 fecal samples were generated and deposited in GenBank (Table 1). The VP2 gene sequences for some of these strains have been previously reported [31]; the VP1 region and the NS genes were obtained here to complete the coding region (Table 1). According to the sequence at codon 426, 10 sequences belonged to CPV-2a and 29 to CPV-2c. The 4269 nt sequence of one sample (370) showed double peaks in several positions in the chromatogram, suggesting the existence of a mixed viral population.

To gain insight into the variability of CPV strains in sample 370, we performed deep sequencing using Illumina technology and obtained ∼206 million high-quality DNA bases with a median coverage of 44×10^3^ sequence reads per base.

The deep-sequencing profile of the CPV coding region was obtained and base frequencies were further analyzed at each nucleotide position. Most nucleotide positions (4240) showed only 1 base at high frequency (Dataset S1). Two nucleotides were observed at high frequency at 29 positions of the 4269 nt coding sequence, which indicated that the sample contained the genomes of two different strains (Table 2). The higher-frequency bases ranged from 51.5 to 62.0% and belonged to a CPV-2c strain; the lower-frequency bases ranged from 37.9 to 48.4% and belonged to a CPV-2a strain (Table 2). The sequences of the CPV-2c (370-2c) and CPV-2a (370-2a) strains contained in the sample were inferred using this deep-sequencing data.

**Table 2:** Deep-sequencing profile of the distinguishing nucleotides between CPV-2a (KM457141) and CPV-2c (KM457142) strains in sample 370.

Nucleotide position	2a	2c	Base frequency (%)				Coverage^a^
			A	T	C	G	
81	A	G	43.3			56.6	50332
342	C	T		57.9	41.9		53834
516	A	G	42.4			57.6	61039
921	A	G	44.0			56.0	48798
1062	A	G	43.1			56.9	49914
1098	A	G	42.6			57.4	55190
1173	C	T		57.3	42.7		55490
1542	C	T		56.5	43.4		56543
1584	T	C		43.1	56.6		58577
1714	A	G	43.0			57.0	46577
1875	A	G	44.0			56.0	45344
1975	T	C		43.2	56.7		58761
2059	A	G	46.8			53.3	45919
2063	G	A	52.6			47.4	44202
2085	G	A	51.5			48.4	42972
2086	A	G	47.9			52.1	43519
2432	G	A	61.2			38.7	22895
2550	G	A	62.0			37.9	25231
2574	T	A	61.3	38.6			25386
2817	C	T		60.0	40.0		35060
3246	C	T		58.7	41.3		47626
3314	A	T	42.4	57.6			48262
3345	C	T		58.3	41.7		43181
3484	A	T	41.4	58.5			45590
3485	T	A	58.7	41.3			45259
3790	A	G	41.7			58.2	48569
3792	T	A	58.1	41.7			47409
3832	G	A	59.4			40.6	46377
4266	C	T		57.1	42.8		3826

a Coverage: number of times a nucleotide is read during the sequencing process.

### Recombination analysis

**Figure 1:**
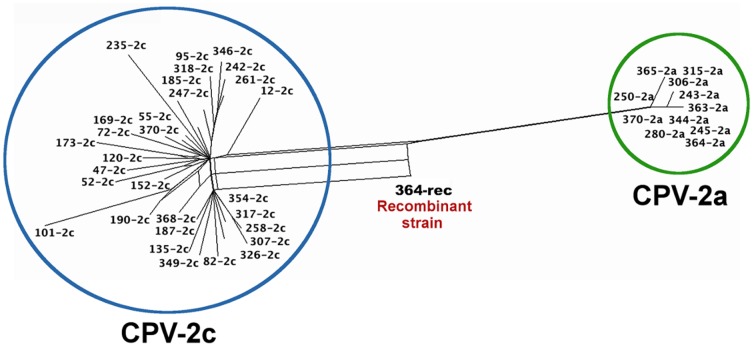
CPV recombination network. The phylogenetic network was generated from alignment of 42 complete CPV genome sequences. There are two main clades encompassing CPV-2c and CPV-2a strains (circles), and an edge (split) that reveals a possible recombination event involving strain 364-rec (KM457139). The Phi test for recombination was significant at p = 0.003.

Because recombination events may lead to conflicting tree topologies in phylogenetic studies, our first approach was to reconstruct a phylogenetic network to explore the ancestral relationships of the CPV strains. The network showed two distinct clusters, one composed of CPV-2c strains and the other of CPV-2a strains (Fig. 1). The co-infecting strains 370-2c and 370-2a grouped with their corresponding CPV-2a and CPV-2c clusters, respectively.

The Uruguayan CPV-2c and CPV-2a clusters were separated by 24 nt substitutions that were constant within each strain but differed between them. These nucleotide changes produced 1 aa difference in NS1, 1 aa difference in the VP1 unique region, and 4 aa differences in VP2 (Table 3). Strains of the CPV-2c cluster were quite variable, with 101 nt and 34 aa differences between strains. The CPV-2a strains had much lower variability, with differences in only 5 nt, four of which were synonymous and one of which was non-synonymous.

**Table 3:** Nucleotide and amino acid differences along the coding sequences of all the Uruguayan CPV-2c and CPV-2a strains, and recombinant 364-rec strain (KM457139).

ORF	NS1								VP1				VP1/VP2											
Nucleotide position	81	342	516	1173	1542	1714	1875	1975	2063	2085	2086	2432	2550	2574	2817	3246	3314	3345	3484	3485	3790	3792	3832	4266
Amino acid identity and position	S27	C114	K172	G391	I514	K572E	Q625	L659	Int^a^	Int^a^	Int^a^	R140K	Q12	A20	T101	Y244	Y267F	H277	I324Y	I324Y	N426E	N426E	A440T	Y584
**2a**	A	C	A	C	C	A	A	T	G	G	A	G	**G**	**T**	**C**	**C**	**A**	**C**	**A**	**T**	**A**	**T**	**G**	**C**
**364-rec**	**G**	**T**	**G**	**T**	**T**	**G**	**G**	**C**	**A**	**A**	**G**	**A**	**G**	**T**	**C**	**C**	**A**	**C**	**A**	**T**	**A**	**T**	**G**	**C**
**2c**	**G**	**T**	**G**	**T**	**T**	**G**	**G**	**C**	**A**	**A**	**G**	**A**	A	A	T	T	T	T	T	A	G	A	A	T

a VP1 Intron. Nucleotides in bold represent identical sequences. Amino acids before and after positions correspond to the CPV-2a and CPV-2c strains, respectively.

Reticulated branches between the CPV-2c and CPV-2a clusters showed evidence (p = 0.03) of a possible recombination event in the strain from sample 364 (hereafter denoted as 364-rec) (Fig. 1). Information regarding the pattern of the 364-rec was obtained using RPD software: the MaxChi, Chimaera, and 3Seq algorithms detected a putative recombination breakpoint at positions 2437 (p = 6.1×10^−4^), 2543 (p = 2.1×10^−3^), and 2542 (p = 5.2×10^−4^), respectively. The breakpoint was within a constant 117-nt region between the last CPV-2c marker (position 2432) and the first CPV-2a marker (position 2550). The breakpoint region was located between the end of the VP1 unique coding region and the beginning of the VP2 gene (Table 3).

### Non-recombinant phylogenies

**Figure 2:**
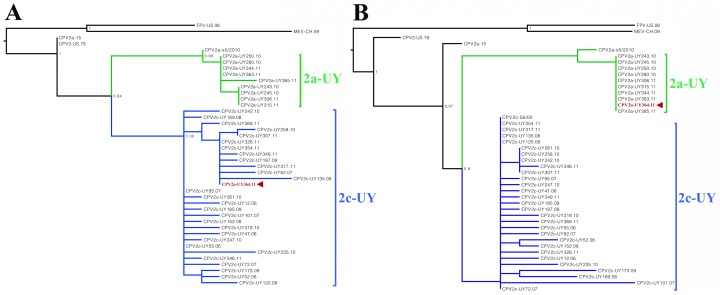
Phylogenetic incongruence analysis. Phylogenetic reconstructions were performed with non-recombinant fragments of the 4269-bp CPV coding region containing nt 1–2500 (A), and nt 2501–4269 (B). The 364-rec strain (arrowhead) associates with both the CPV-2c (A) and CPV-2a (B) clades. 2a-UY: Uruguayan CPV-2a clade; 2c-UY: Uruguayan CPV-2c clade; FPV-US.06: feline panleukopenia virus (EU659115); MEV-CH.09: mink enteritis virus (FJ592174); CPV2-US.79: Canine parvovirus type 2 (M38245); CPV2a-15 United States 2a isolate (M24003); CPV2a-s5/2010 Chinese 2a isolate (KF638400.1). CPV2c-56/00 Italian 2c isolate. CH: China; US: United States; UY: Uruguay.

To further analyze the recombination event that occurred between CPV-2c and CPV-2a, a phylogenetic incongruence analysis was performed using recombinant and non-recombinant sequences. Genome sequences were divided into two alignments around the site of the breakpoint (nt 2500), and separate phylogenetic trees for each dataset were constructed (Fig. 2). The phylogenetic trees constructed with non-recombinant fragments of the NS and VP2 genes corroborated the chimeric pattern found in the genome of the 364-rec strain. The left fragment (NS gene) of the recombinant strain was derived from a CPV-2c strain, and the right fragment, including the VP2 gene, was from a CPV-2a strain (Table 3). The 364-rec strain was not associated with CPV-2 strains (vaccine type), MEV, or FPV, indicating that it was not derived from any of these strains (Fig. 2).

### Analysis of the recombinant strain

To confirm the recombination event, we cloned a single 4269-bp amplicon that included the full-length coding region of the putative recombinant strain. Both ends (approximately 800 bp) of the CPV fragment of twenty clones were sequenced for preliminary classification using positions 81, 342, and 516 (5′ terminus) and positions 4266, 3832, 3792, and 3790 (3′ terminus) as markers for CPV-2a and CPV-2c identification. From this partial sequencing, 15 clones were identified as recombinant, but 5 clones were non-recombinant and corresponded to a typical CPV-2a strain. The complete sequence of three recombinant clones and one CPV-2a clone confirmed the classifications based on partial terminal sequences. These findings indicate that sample 364 is a mixture of a recombinant strain (364-rec) and a non-recombinant CPV-2a strain (364-2a).

To characterize the CPV strain structure of sample 364 with a greater degree of accuracy, we deep sequenced this sample and obtained ∼363 million high-quality DNA bases with a median coverage of 93×10^3^ sequence reads per base (Dataset S2). Deep sequencing confirmed that sample 364 includes a major recombinant strain and a CPV-2a strain. The analysis of base frequencies revealed that 364-rec constituted 86.7% of the viral population and 364-2a constituted the remaining 13.3% (Table 4). The strains differed at 18 nt in the recombinant region from position 1 to position 2432; 364-2a had the typical CPV-2a nucleotide residues, and 364-rec had the CPV-2c residues at their corresponding frequencies (Table 4). There was one difference after the breakpoint (nt 2907), which may correspond to a single-nucleotide polymorphism in the 2a homologous region of both strains; the 364-2a strain had a C and 364-rec had a T at this position (Table 4). The analysis of the reconstructed sequences revealed that the recombinant strain sequence obtained by deep sequencing was identical to the sequences obtained by traditional Sanger sequencing and that the 364-2a strain clustered with CPV-2a (Fig. 1).

**Table 4:** Deep-sequencing profile of the distinguishing nucleotides between CPV-2a (KM457143) and 364-rec recombinant (KM457139) strains in sample 364.

Nucleotide position	2a	364-rec^a^	Base frequency (%)				Coverage^b^
			A	T	C	G	
81	A	G	12.2			87.6	83302
105	C	T		87.0	13.0		92516
342	C	T		87.1	12.8		91505
516	A	G	13.0			87.0	119020
1053	T	A	87.6	12.3			105033
1062	A	G	12.5			87.5	105127
1098	A	G	13.0			87.0	115943
1173	C	T		86.6	13.3		109654
1377	C	T		89.0	11.0		129477
1542	C	T		86.4	13.6		123467
1714	A	G	13.2			86.8	102937
1875	A	G	13.3			86.7	92231
1975	T	C		12.2	87.7		112933
2059	A	G	15.5			85.5	79770
2063	G	A	84.4			15.6	75469
2085	G	A	83.8			16.1	64731
2086	A	G	15.9			84.1	65791
2432	G	A	86.9			13.1	39011
2433–2549: breakpoint region^c^							
2907^d^	C	T		89.0	11.0		66466

a The left fragment (NS gene) of the 364-rec strain was derived from a CPV-2c strain, and the right fragment was from a CPV-2a strain.

b Coverage: number of times a nucleotide is read during the sequencing process.

c The recombination breakpoint in the 364-rec recombinant strain falls in a 117-nt region between two constant parental markers (positions 2433 and 2549).

d The difference in the 2907 position corresponds to a single-nucleotide polymorphism in the 2a homologous region of the co-infecting strains.

## Discussion

Our findings indicate that Uruguayan CPV-2c and CPV-2a are two divergent phylogenetic groups that are differentiated by both VP1/VP2 and NS sequences. This variability supports the hypothesis, previously proposed based on the VP2 gene analysis, that Uruguayan CPV-2c and CPV-2a evolved independently from European and Asiatic strains [30], [31]. The level of divergence between CPV-2c and CPV-2a strains is relatively high and includes 24 consistent nucleotide substitutions and 6 aa changes (Table 3). Four of these amino acid variations are located in the highly variable D–H loop of VP2, but synonymous changes are distributed along the complete coding region. The CPV-2c strains are more genetically variable, presumably because of the longer presence of CPV-2c in South America, which likely dates back to at least 2005 when CPV strains of European origin are thought to have been introduced to the continent [31]. The CPV-2a strains are nearly identical, supporting the theory that this variant was recently introduced to South America from Asia during 2010 [31]. The Asiatic origin of CPV-2a is here confirmed by the close phylogenetic relationship between the NS and VP sequences of Uruguayan strains and the Chinese isolate CPV-2a-s5/2010 (Fig. 2). European origin of the 2c variants could not be fully confirmed because there are not European NS sequences available for comparison.

The existence of a CPV population formed by two divergent variants of different geographic origin is quite extraordinary and offers the unique opportunity to analyze the interaction dynamics of CPV-2c and CPV-2a and to study recent co-infection and recombination events in the field.

The present study describes a case of co-infection with divergent CPV-2a and CPV-2c strains and characterizes the genetic structure of the co-infecting strains using deep-sequencing technology. To our knowledge, this is the first study to apply this technology to explore the diversity of canine parvovirus. Deep-sequencing proved to be a powerful tool for determining the strains included in a sample and inferring the complete sequences and frequencies of the co-infecting strains, providing new data about intra-host strain dynamics.

Cases of co-infections in dogs are surprising, as CPV causes acute infections that are cleared in a few weeks, with no residual replicating viruses in surviving animals [41], [42]. Entry of the second variant must occur during the brief period prior to an immune response, which could certainly be facilitated by the high prevalence of the virus in high-density dog populations. Our results emphasize the importance of co-infection as a source of genetic complexity in parvovirus and the need to consider co-infecting strains when analyzing CPV pathogenicity; CPV variants may have differences in their pathogenicity [43], [44] and thus their co-existence in the same host could potentially influence disease symptoms.

The detection of co-infecting strains is an indicative that genetic recombination might be occurring in the population. Recombination has played a significant role in the evolutionary process and in the generation of the global diversity of parvoviruses. Available genetic data provide evidence of natural recombination among porcine, mink, and several rodent parvoviruses [45]. Recombination in CPV was recently described between field and vaccine strains, and with related carnivore parvovirus [25]–[27]. The present study is the first to describe recombination among circulating CPV-2 strains and to reveal that recombinant strains are able to infect and spread in dogs. Moreover, we established that this recombination was relatively recent and likely occurred in the ∼18-month period that CPV-2a and CPV-2c strains are thought to have co-existed in Uruguay [31]. This result indicates that co-circulating strains may recombine in a relatively short time, even if they are of different antigenic types and exhibit marked genetic divergence.

In our study, we did not detect any recombination between CPV-2a/c and the vaccine (CPV-2) strains, even though several dogs became ill after receiving the vaccine. In fact, the dog from which the 364-rec strain was isolated from (a 3-month old Saint Bernard female owned by a city resident) had received three CPV-2 type vaccine doses just 3 weeks before getting ill.

Another intriguing aspect of the recombinant strain is that it occurred together with a parental CPV-2a strain. This co-infection was not detected with traditional Sanger sequencing of the PCR fragments, and the virus was initially considered a single recombinant strain. However, molecular cloning and deep sequencing of the amplicons revealed the minor presence of a CPV-2a strain (13.3–25% of the viral population).

Several observations suggest that it is more likely that this dog was independently infected by 364-rec and a CPV-2a strain than that the recombination process occurred within this dog. First, recombination is a sporadic event, and the probability of identifying the individual host in which it occurred is very low. Moreover, the deep-sequencing profile revealed one single-nucleotide polymorphism in the CPV-2a region of the recombinant and non-recombinant strains, which suggests that these sequences did not have the same origin. Finally, deep sequencing also showed that the 364 sample did not contain the CPV-2c variant. It is worth noting that the absence of CPV-2c in this sample supplies additional evidence that the recombinant DNA genome is not artificially produced from co-infecting CPV-2a and CPV-2c genomes by PCR amplification.

Recombination breakpoints in naturally occurring ssDNA viruses are generally not randomly distributed. Most identified ssDNA virus breakpoints occur within intergenic regions or near gene boundaries, because this tends to be less deleterious to the organism [46]. In our study, the novel recombinant strain arose from inter-genotypic recombination between CPV-2c and CPV-2a strains within the VP1/VP2 gene boundary. This recombination pattern supports the idea that the VP2 gene is selected for in the natural host of the virus and tends to be transferred as an intact unit to avoid disrupting favorable intra-protein interactions [47].

Similar recombination patterns have been identified between CPV and FPV or MEV, viruses that are considered host variants of the same species [26], [27]. In both cases, the recombination breakpoint falls in a 397-nt region between two constant parental markers (positions 2355 and 2753), which encompasses the 117-nt breakpoint region detected in our study. Interestingly, the VP2 gene of the known recombinant strains appears to be derived from the viral strain that usually infects that host: the recombinant virus isolated from cats carries the VP2 gene of FPV, and the recombinant strain isolated from mink has the VP2 gene of MEV. This supports the hypothesis that selection promotes capsid gene transfer from one genetic background to another but retains the adaptation of VP2 to a particular environment or host. In our case, the recombinant strain carried the VP2 gene of CPV-2a, suggesting that CPV-2a has a higher fitness in the present Uruguayan dog population than CPV-2c, maybe as a consequence of a higher natural immunity developed against CPV-2c due to its longer presence in the country. This hypothesis is supported by epidemiological observations that CPV-2a appears to be replacing CPV-2c in the population of Uruguayan dogs [30], [31]. Moreover, the higher frequency of the recombinant strain than the CPV-2a strain in the co-infected dog in this study raises the possibility that the recombinant has a replicative advantage. It is assumed that if such adaptive recombination occurs, the resulting recombinant should increase in prevalence to become a detectable circulating form [46]. The biological significance of the recombinant strain remains to be studied and it would be interesting to establish how its fitness compares to that of the parental viruses in tissue culture and *in vivo* models. As the recombinant strain appears to have emerged very recently, it is possible that it has undergone very limited expansion in the population.

Recombination in ssDNA viruses has been proposed to arise during DNA synthesis and likely involves a number of different processes that are still poorly characterized [46]. Parvoviruses replicates by a continuous unidirectional strand-displacement mechanism, known as rolling hairpin replication [48]. This mechanism is a variation of the rolling circle strategy used by small circular molecules, but has been adapted for the amplification of the single-stranded linear genome of parvoviruses using terminal hairpins as primers for complementary strand synthesis [49]. During the rapid DNA synthesis, the host replicative machinery may switch between viral templates and produces recombinant molecules [46]. Analysis of the 117-nt region where the breakpoint occurred in the 364-rec strain revealed the presence of a unique homopolymeric tract (a tract of 13 thymidines that codes for four Lys residues in the exclusive VP1 region). These sequence motifs are associated with pausing of DNA synthesis, which is thought to be important for promoting template switching [50]. The correlation between the homopolymeric tract and the recombination hotspot is particularly interesting, as no single sequence has yet been suggested to promote recombination in parvovirus. Secondary and tertiary DNA structures may also influence premature detachment of replication machinery, but this is a challenging topic that needs further study.

Taken together, our findings indicate that co-infection and recombination are currently occurring between circulating strains in natural populations of CPV, indicating that both may be relevant forces in the generation of viral diversity and the emergence of new genotypes. Rapid genomic evolution by genetic interchange may be regulated by restricting recombination to particular hotspots in the genome that favor the interchange of the entire VP2 gene. As most studies only analyze VP2 sequences, the detection of recombinant strains is severely hindered, highlighting the benefits of characterizing complete genomes rather than focusing on individual components. It is also becoming increasingly clear that studying only the consensus sequence of DNA viruses insufficiently summarizes the diversity of the viral population. In this sense, our study underscores the importance of deep-sequencing analysis to provide a better understanding of CPV molecular diversity. It is expected that this technology will become a useful tool for future studies of parvoviruses.

## Supporting Information

Figure S1 **Amplicons and primers used in the molecular cloning and deep-sequencing analyses.** Sequences of all primer used in this study are included below. The asterik indicates primers that were used for Sanger sequencing of the full-length genome.
(PPTX)

Dataset S1 **Deep-sequencing profile in sample 370; the two PCR amplicons that together span the entire CPV coding sequence (4269 nt) include a CPV-2a and CPV-2c strains (distinguishing nucleotides between both strains are highlighted in yellow).**
(XLSX)

Dataset S2 **Deep-sequencing profile in sample 364; the two PCR amplicons that together span the entire CPV coding sequence (4269 nt) include a CPV-2a and a the 364-rec recombinant strain) CPV-2c strains (distinguishing nucleotides between both strains are highlighted in yellow, the breakpoint region in red, the 2907 SNP in green and the homopolymeric tract in blue).**
(XLSX)
