# Supplementary material for: Phylogenetic and Genome-Wide Deep-Sequencing Analyses of Canine Parvovirus Reveal Co-Infection with Field Variants and Emergence of a Recent Recombinant Strain
Source: PLoS One. 2014 Nov 3;9(11):e111779. doi: 10.1371/journal.pone.0111779 (PMC4218814; doi:10.1371/journal.pone.0111779)
Supplement: Figure S1 — Amplicons and primers used in the molecular cloning and deep-sequencing analyses. Sequences of all primer used in this study are included below. The asterik indicates primers that were used for Sanger sequencing of the full-length genome. (PPTX) [file pone.0111779.s001.pptx]

## Slide 1
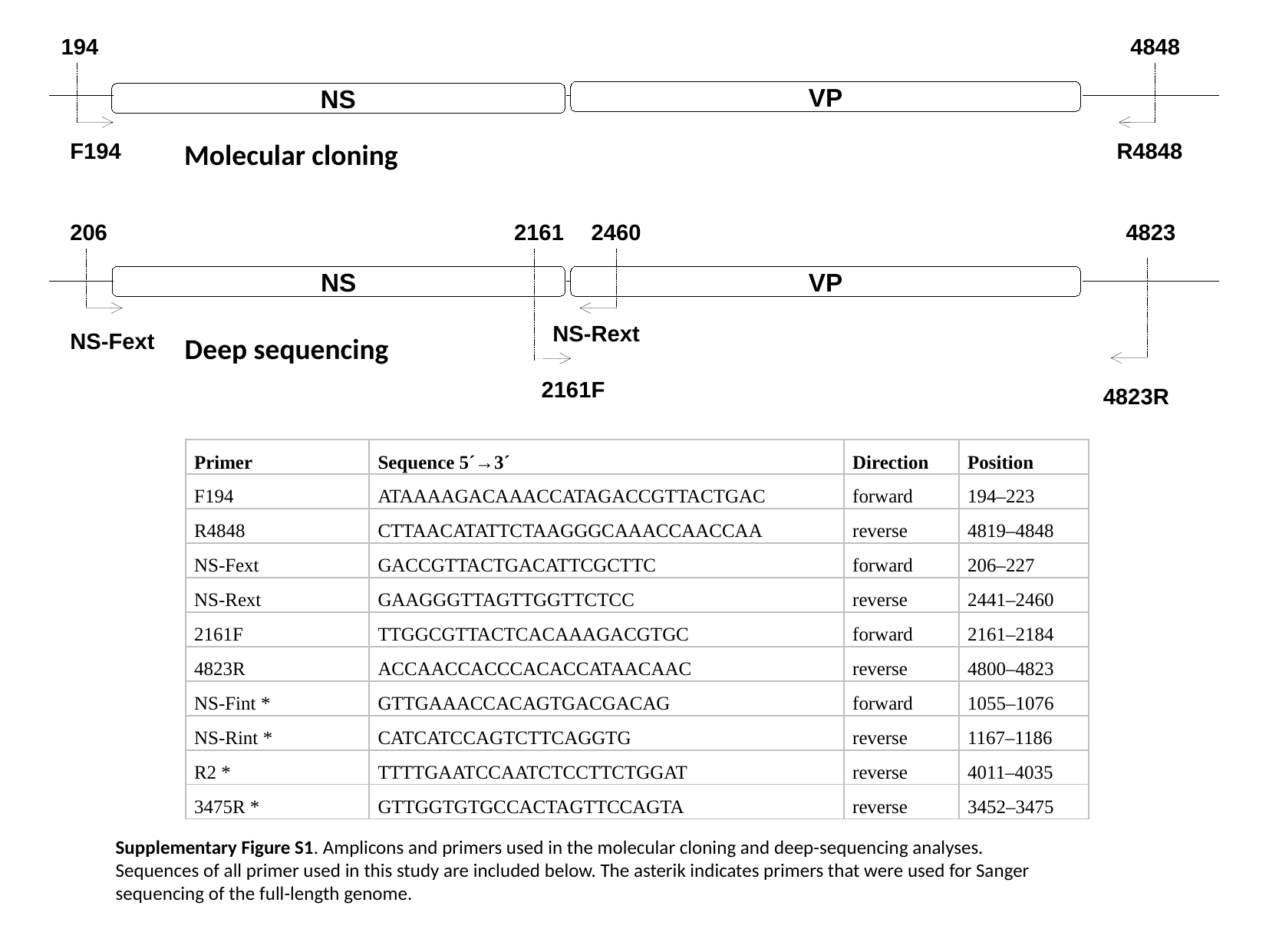

194
4848
VP
NS
Molecular cloning
F194
R4848
206
2161
2460
4823
NS
VP
NS-Rext
NS-Fext
Deep sequencing
2161F
4823R
| Primer | Sequence 5´→3´ | Direction | Position |
| --- | --- | --- | --- |
| F194 | ATAAAAGACAAACCATAGACCGTTACTGAC | forward | 194–223 |
| R4848 | CTTAACATATTCTAAGGGCAAACCAACCAA | reverse | 4819–4848 |
| NS-Fext | GACCGTTACTGACATTCGCTTC | forward | 206–227 |
| NS-Rext | GAAGGGTTAGTTGGTTCTCC | reverse | 2441–2460 |
| 2161F | TTGGCGTTACTCACAAAGACGTGC | forward | 2161–2184 |
| 4823R | ACCAACCACCCACACCATAACAAC | reverse | 4800–4823 |
| NS-Fint \* | GTTGAAACCACAGTGACGACAG | forward | 1055–1076 |
| NS-Rint \* | CATCATCCAGTCTTCAGGTG | reverse | 1167–1186 |
| R2 \* | TTTTGAATCCAATCTCCTTCTGGAT | reverse | 4011–4035 |
| 3475R \* | GTTGGTGTGCCACTAGTTCCAGTA | reverse | 3452–3475 |
Supplementary Figure S1. Amplicons and primers used in the molecular cloning and deep-sequencing analyses. Sequences of all primer used in this study are included below. The asterik indicates primers that were used for Sanger sequencing of the full-length genome.
